# Supplementary material for: Comparative efficacy of robot-assisted therapy associated with other different interventions on upper limb rehabilitation after stroke: A protocol for a network meta-analysis
Source: PLoS One. 2025 Jan 28;20(1):e0304322. doi: 10.1371/journal.pone.0304322 (PMC11774368; doi:10.1371/journal.pone.0304322)
Supplement: S1 File — (DOCX) [file pone.0304322.s002.docx]

Supplementary Material 1: A draft search strategy

1. **CINAHL**

(SU =stroke* OR SU =apoplexy* OR SU =cerebral vascular accident OR SU =brain vascular accident OR SU =cerebral vascular disorders OR SU =brain vascular disorders OR SU =intracranial hemorrhage OR SU =cerebral hemorrhage OR SU =brain hemorrhage OR SU =subarachnoid hemorrhage OR SU =cerebral infarction OR SU =brain infarction OR SU =cerebral ischemia OR SU =brain ischemia) AND (SU =upper limb OR SU =upper extremity OR SU =arm OR SU =hand OR SU =shoulder OR SU =elbow OR SU =forearm OR SU =finger OR SU =wrist) AND (SU =robot* OR SU =robot-assisted OR SU =exoskeleton) AND (SU =randomized controlled trial OR SU =trial).

1. **PubMed**

(“stroke*” OR “apoplexy*” OR “cerebral vascular accident” OR “brain vascular accident” OR “cerebral vascular disorders” OR “brain vascular disorders” OR “intracranial hemorrhage” OR “cerebral hemorrhage” OR “brain hemorrhage” OR “subarachnoid hemorrhage” OR “cerebral infarction” OR “brain infarction” OR “cerebral ischemia” OR “brain ischemia”) AND (“upper limb” OR “upper extremity” OR “arm” OR “hand” OR “shoulder” OR “elbow” OR “forearm” OR “finger” OR “wrist”) AND (“robot*” OR “robot-assisted” OR “exoskeleton”) AND (“randomized controlled trial” OR “trial”).

1. **Web of Science**

(TS =stroke* OR TS =apoplexy* OR TS =cerebral vascular accident OR TS =brain vascular accident OR TS =cerebral vascular disorders OR TS =brain vascular disorders OR TS =intracranial hemorrhage OR TS =cerebral hemorrhage OR TS =brain hemorrhage OR TS =subarachnoid hemorrhage OR TS =cerebral infarction OR TS =brain infarction OR TS =cerebral ischemia OR TS =brain ischemia) AND (TS =upper limb OR TS =upper extremity OR TS =arm OR TS =hand OR TS =shoulder OR TS =elbow OR TS =forearm OR TS =finger OR TS =wrist) AND (TS =robot* OR TS =robot-assisted OR TS =exoskeleton) AND (TS =randomized controlled trial OR TS =trial).

1. **MEDLINE**

(stroke* OR apoplexy* OR cerebral vascular accident OR brain vascular accident OR cerebral vascular disorders OR brain vascular disorders OR intracranial hemorrhage OR cerebral hemorrhage OR brain hemorrhage OR subarachnoid hemorrhage OR cerebral infarction OR brain infarction OR cerebral ischemia OR brain ischemia) AND (upper limb OR upper extremity OR arm OR hand OR shoulder OR elbow OR forearm OR finger OR wrist) AND (robot* OR robot-assisted OR exoskeleton) AND (randomized controlled trial OR trial).

1. **EMBASE**

(stroke* OR apoplexy* OR cerebral vascular accident OR brain vascular accident OR cerebral vascular disorders OR brain vascular disorders OR intracranial hemorrhage OR cerebral hemorrhage OR brain hemorrhage OR subarachnoid hemorrhage OR cerebral infarction OR brain infarction OR cerebral ischemia OR brain ischemia) AND (upper limb OR upper extremity OR arm OR hand OR shoulder OR elbow OR forearm OR finger OR wrist) AND (robot* OR robot-assisted OR exoskeleton) AND (randomized controlled trial OR trial).

1. **CNKI**

( SU=卒中 OR SU=脑梗死 OR SU=脑出血 OR SU=脑血管疾病 OR SU=脑血管意外 OR SU=蛛网膜下腔出血) AND ( SU=上肢 OR SU=手臂 OR SU=手 OR SU=肩 OR SU=肘 OR SU=前臂 OR SU=手腕 OR SU=手指) AND ( SU=机器人 OR SU=外骨骼) AND ( SU=随机对照试验 OR SU=临床试验)

1. **Wanfang Database**

( “卒中” OR “脑梗死” OR “脑出血” OR “脑血管疾病” OR “脑血管意外” OR “蛛网膜下腔出血”) AND ( “上肢” OR “手臂” OR “手” OR “肩” OR “肘” OR “前臂” OR “手腕” OR “手指”) AND ( “机器人” OR “外骨骼”) AND ( “随机对照试验” OR “临床试验”)
