# Supplementary material for: Comparative efficacy of robot-assisted therapy associated with other different interventions on upper limb rehabilitation after stroke: A protocol for a network meta-analysis
Source: PLoS One. 2025 Jan 28;20(1):e0304322. doi: 10.1371/journal.pone.0304322 (PMC11774368; doi:10.1371/journal.pone.0304322)
Supplement: S1 Table — (DOCX) [file pone.0304322.s003.docx]

Data extraction form

| Authors (year) | Age (years old) | Sex  (male/female) | Sample size | Type of disease | Intervention | Control | Frequency and duration of intervention | Outcomes and measurements | Scores of outcomes |
| --- | --- | --- | --- | --- | --- | --- | --- | --- | --- |
|  |  |  |  |  |  |  |  |  |  |
|  |  |  |  |  |  |  |  |  |  |
|  |  |  |  |  |  |  |  |  |  |
|  |  |  |  |  |  |  |  |  |  |
|  |  |  |  |  |  |  |  |  |  |
|  |  |  |  |  |  |  |  |  |  |
|  |  |  |  |  |  |  |  |  |  |
|  |  |  |  |  |  |  |  |  |  |
