# Supplementary material for: Comparative efficacy of robot-assisted therapy associated with other different interventions on upper limb rehabilitation after stroke: A protocol for a network meta-analysis
Source: PLoS One. 2025 Jan 28;20(1):e0304322. doi: 10.1371/journal.pone.0304322 (PMC11774368; doi:10.1371/journal.pone.0304322)
Supplement: Attachment — Submitted filename: 2.26 Response to Reviewers.docx [file pone.0304322.s004.docx]

Response to Reviewers

Dear Editor and reviewers,

Thank you for your kind advice and providing us the chance to revise our manuscript entitled “Comparative efficacy of robot-assisted therapy associated with other different interventions on upper limb rehabilitation after stroke: a protocol for a network meta-analysis” (ID: PONE-D-23-41381).

Following the editor’s guidance, we ensure that our manuscript meets PLOS ONE&’s style requirements. We provided the correct grant numbers (HXDZ21003).

Following the suggestion, we have revised the manuscript point by point according to the comments. If we did not get what you mean, please give us another chance to make modifications. The details are as follows.

Responses to reviewer 1:

1. From a clinical point of view, the authors neglect the major concept of “opportunity window”; for recovery, which would strongly bias results. I suggest either they focus on the first 3/6 months after the event, or conduct a separate analysis for the early/late subacute and chronic phase.

Reply: Thanks for the kind advice. We have reviewed literature and conducted a discussion. We decided that the types of participants focused on the first six months after event and revised the inclusion criteria. (page 5, line 20)

2. The authors pool together very different types of brain lesions, ranging from SH to intracranial hemorrhage and hemorrhagic/ischemic stroke. They need to refocus the etiology to be consistent with the planned title.

Reply: Thanks for the kind advice. The target disease of this meta-analysis is stroke. Based on our previous search, some studies on intracranial hemorrhage, hemorrhagic/ischemic stroke, cerebral vascular accident and subarachnoid hemorrhage also target stroke. In addition, in researches on robot-assisted rehabilitation, most studies focused on all types of stroke patients, rather than just one type of stroke. Therefore, we considered all types of stroke when formulating the retrieval strategy (page 7, lines 4-7), to avoid obtaining insufficient literature in future literature searches.

3. Primary outcome measures are heterogeneous. The authors just report a few among the most commonly used scales for body integrity and function (see ICF), but they need to report all the outcome measures (primary and secondary) which they plan to include.

Reply: Thanks for the kind advice. We searched for literature related to this meta-analysis topic and supplemented the outcome measures that used to measure upper limb function and activities of daily living. (page 6, lines 17-22)

4. The authors need also to provide a clear list of the associated interventions they plan to include: for example, they briefly mention NIBS. In this case, they should detail which NIBS (rTMS, tDCS, tACS?) and possibly the protocol they will consider. The same holds true for other interventions, with a report of dosing and intensity. This may push up heterogeneity - the authors will need to discuss how they plan to manage this.

Reply: Thanks for the kind advice. Based on our understanding, we described the possible interventions that may be considered in types of interventions section (page 6, lines 4-7). Based on clinical work and literature review, the dosage and intensity will be limited to once a day and at least three times a week (page 6, lines 8-9). If we did not get what you mean, please give us another chance to make modifications.

5. From a methodological point of view, the authors do not mention how they will deal with direct, indirect and mixed comparisons, as well as assess inconsistencies.

Reply: Thanks for the kind advice. We have described the statistical methods in more detail. For direct, indirect and mixed comparisons, as well as assess inconsistencies, we added relevant statistical analysis methods. (page 9, lines 12-22; page 10, lines1-4)

6. I would also suggest attaching as supplementary the data extraction form.

Reply: Thanks for the kind advice. We attached as supplementary the data extraction form (page 8, line 8).

7. A thorough revision by a native speaker is mandatory

Reply: Thanks for the advice. we invited a scholar, who is a native English speaker and also with technical knowledge of this subject, to assist us during this revision and help us improve the quality of the writing.

Responses to reviewer 2:

1. the authors stated that there is no research comparing which rehabilitation program combined with robot-assisted therapy has the best effect. This is a very strong statement, especially given the large number of research published every day. Therefore, I recommend that authors inform in the introduction how they arrived at this information or improve the text in this section.

Reply: Thanks for the kind advice. After discussion and literature review, we agree that such a statement is indeed inappropriate. Therefore, we revised this sentence in the introduction section. (page 5, lines 7-9)

2. In the statistical analysis section, the authors need to better justify the techniques to be used, as the selection criteria are not clear.

Reply: Thanks for the kind advice. We have described the statistical methods in more detail. For direct, indirect and mixed comparisons, we added relevant statistical analysis methods. (page 9, lines 12-22; page 10, lines1-4)

3. The authors also did not inform whether they will make the analyzed data available, this is important, as it guarantees the reproducibility of the research.

Reply: Thanks for the advice. Because this meta-analysis will be based on published data and no new data will be generated, we did not inform whether we will make the analyzed data available. The data we will extract and analyze can be obtained through reasonable requests to the corresponding authors. (page 12, lines 2-4)

4. The authors made the following statement: “To date, no studies have explored which rehabilitation programs are most effective in combination with robot-assisted therapy”, but they do not say how they reached this conclusion. I recommend that the authors inform how they reached this conclusion or improve the text.

Reply: Thanks for the kind advice. After discussion and literature review, we agree that such a statement is indeed inappropriate. Therefore, we revised this sentence in the introduction section. (page 11, lines 8-10)

5. The discussion is redundant, as some parts have already been highlighted very well in the introduction. My recommendation is that the authors discuss the importance of their study and how it can be used, for example, in the formulation of public health policies.

Reply: Thanks for the kind advice. We added some discussion about the importance of this study. (page 11, lines 11-19)

6. Authors need to describe where all data underlying the results will be made available when the study is completed.

Reply: Thanks for the advice. We revised the Data Availability section, and when the study is completed, all relevant data underlying the results will be made available through reasonable requests to the corresponding authors. (page 12, lines 2-4)
